# Apatinib attenuates phenotypic switching of arterial smooth muscle cells in vascular remodelling by targeting the PDGF Receptor‐β

**DOI:** 10.1111/jcmm.15623

**Published:** 2020-07-22

**Authors:** Wenchao Shao, Xiaoguang Li, Jiangtong Peng, Siyuan Fan, Minglu Liang, Kai Huang

**Affiliations:** ^1^ Clinic Center of Human Gene Research Union Hospital Tongji Medical College Huazhong University of Science and Technology Wuhan China; ^2^ Department of Cardiology Union Hospital Tongji Medical College Huazhong University of Science and Technology Wuhan China

**Keywords:** Apatinib, PDGF Receptor‐β, vascular smooth muscle cell phenotypic switching

## Abstract

Apatinib (YN968D1) is a small‐molecule tyrosine kinase inhibitor（TKI）which can inhibit the activity of vascular endothelial growth factor receptor‐2 (VEGFR‐2). It has been reported that apatinib has anti‐tumour effect of inhibiting proliferation and inducing apoptosis of a variety of solid tumour cells, whereas its effect on vascular smooth muscle cells (VSMC) remains unclear. This study investigated the effect of apatinib on phenotypic switching of arterial smooth muscle cells in vascular remodelling. Compared to the vehicle groups, mice that were performed carotid artery ligation injury and treated with apatinib produced a reduction in abnormal neointimal area. For in vitro experiment, apatinib administration inhibited VSMC proliferation, migration and reversed VSMC dedifferentiation with the stimulation of platelet‐derived growth factor type BB (PDGF‐BB).In terms of mechanism, with the preincubation of apatinib, the activations of PDGF receptor‐β (PDGFR‐β) and phosphoinositide‐specific phospholipase C‐γ1 (PLC‐γ1) induced by PDGF‐BB were inhibited in VSMCs. With the preincubation of apatinib, the phosphorylation of PDGFR‐β, extracellular signal‐related kinases (ERK1/2) and Jun amino‐terminal kinases (JNK) induced by PDGF‐BB were also inhibited in rat vascular smooth muscle cell line A7r5. Herein, we found that apatinib attenuates phenotypic switching of arterial smooth muscle cells induced by PDGF‐BB in vitro and vascular remodelling in vivo. Therefore, apatinib is a potential candidate to treat vascular proliferative diseases.

## 1 INTRODUCTION

Arterial balloon dilatation, stent implantation, and vascular bypass transplantation are effective measures for the treatment of acute and chronic vascular obstructive diseases.^1^ Vascular restenosis is a common long‐term complication after revascularization, mainly manifested as intimal hyperplasia which is an important cause of in‐stent restenosis and bridging vessel occlusion.^2^ The main components of neointima are VSMCs that undergo phenotypic transformation.^3^ The existing first‐line therapy cannot effectively inhibit abnormal neointimal hyperplasia; therefore, it is necessary to re‐understand and find drugs that effectively inhibit phenotypic transformation of VSMC.

PDGF‐BB, as a natural ligand of PDGFR‐β, is a potent cellular mitogen and chemoattractant for VSMC, which could activate its downstream signalling pathways and contribute to many biological processes and disease genesis after binding with PDGFR‐β.^4^ For instance, PDGFR‐β signalling pathway and its downstream signalling MAPKs have been demonstrated to have an effect on cell proliferation and migration.^5^ Furthermore, numerous studies have reported that PDGFR‐β‐mediated downstream signalling pathway is dysregulated in a variety of vascular diseases.

Apatinib (C_25_H_27_N_5_O_4_S) is one of the small‐molecule inhibitors with anti‐tumour effect that firstly proved safely and effectively in the treatment of advanced gastric cancer.^6^ A low dose of apatinib optimizes the tumour microenvironment in lung cancer.^7^ Apatinib inhibits extrahepatic bile duct cancer (EBDC) cell proliferation induced by intracellular autocrine VEGF signalling.^8^ Apatinib binds to VEGFR‐2 selectively and inhibits its function (IC_50_ 1nM), as well as inhibits the activity of PDGFR‐β.^9^ Therefore, apatinib is expected to apply to a new field, namely the inhibition of VSMC phenotypic switching in vascular remodelling by targeting the PDGFR‐β. No studies have been reported the relationship between apatinib and VSMC phenotypic switching.

## 2 MATERIALS AND METHODS

### 2.1 Materials

Apatinib was purchased from MedChemExpress. Recombinant human PDGF‐BB was purchased from Corning Incorporated. Antibodies of the total levels and phosphorylation of PDGFR‐β, PLC‐γ1, ERK1/2, JNK and p38‐MAPK were purchased from Cell Signaling Technology. Proliferating cell nuclear antigen (PCNA), anti‐smooth muscle α‐actin (SMα‐actin), anti‐α‐tublin, anti‐Cyclin D1, FITC‐conjugated secondary rabbit antibodies were purchased from Proteintech. Anti‐matrix metalloproteinase‐2 (MMP‐2), anti‐matrix metalloproteinase‐9 (MMP‐9), anti‐SM22α, anti‐caspase3, anti‐Bcl2, anti‐Bax, anti‐P21 and anti‐P27 were purchased from Abcam. EdU kit was purchased from Ribobio. Trypsin, Dulbecco's Modified Eagle's Medium (DMEM) and foetal bovine serum (FBS) were purchased from GIBCO. Apatinib was dissolved in dimethylsulphoxide (DMSO) for studies. DMSO alone was used as vehicle.

### 2.2 Cell and culture

The primary rat VSMCs were isolated from the thoracic aorta of male Sprague‐Dawley(S‐D) rats in an enzymatical way. Cells from passage 3‐6 were used for in vitro experiments. The rat vascular smooth muscle cell line A7r5 was obtained from the ATCC. Cells were cultured in DMEM medium with 10% FBS and serum‐starved for 24 hours before pretreatment of apatinib.

### 2.3 EdU incorporation assay

Before being stimulated with PDGF‐BB (30 ng/mL) or not for 48 hours, VSMCs were seeded into 96‐well plates and pretreated with apatinib or vehicle for 4 hours. EdU Incorporation Assay was performed according to the manufacturer's instructions. Images were photographed by Olympus cellSens Entry.

### 2.4 Wound healing assays

VSMCs were seeded into 6‐well plates and cultured up to 80% density. Cell monolayers were scratched using a 1 mL‐pipette tip. Before the stimulation of PDGF‐BB (30 ng/mL) or not for 48 hours, cells were preincubated with apatinib at various concentrations for 4 hours then cultured in DMEM containing 10% FBS. Cells were viewed using Olympus cellSens Entry; the rate of wound closure was measured with program ImageJ.

### 2.5 Transwell assays

Transwell assays were used to determine cell migration. After pretreatment of apatinib for 4 hours, VSMCs were seeded into the upper chambers, 500 μL DMEM with 10% FBS and PDGF‐BB (30 ng/mL) was placed into the lower chamber. 12 hours later, the migrated cells in the lower chamber were fixed with 4% formaldehyde for 20 minutes and then stained with 0.1% crystal violet for another 20 minutes. The migrated cells were photographed using Olympus cellSens Entry.

### 2.6 Western blotting

VSMCs or cell line A7r5 was cultured in 6‐well plates up to 80% density. After being preincubated with apatinib for 1 or 4 hours, cells were stimulated with PDFG‐BB (30 ng/mL) for certain time. Western blots were performed following the steps as described previously.^10^

### 2.7 Immunofluorescence analysis

Immunofluorescence was performed to evaluate VSMC dedifferentiation. VSMCs were cultured in 24‐well plates and pretreated with vehicle or apatinib at the maximum concentration (200 nmol/L) for 4 hours and stimulated with PDGF‐BB (30 ng/mL) for 48 hours. Cells were fixed in 4% formaldehyde for 20 minutes and blocked with 1% BSA for 30 minutes. Cells were stained with antibody SMα‐actin (1:100) overnight at 4°C and incubated with FITC‐conjugated fluorescence secondary antibody (1:500) for 2 hours at 37°C. Nuclei were stained with DAPI for 15 minutes at 37°C. Cells were photographed using Olympus cellSens Entry. For sections harvested from animal model, the sections were incubated with primary antibody SMα‐actin (1:100) overnight at 4°C and incubated with FITC‐conjugated fluorescence secondary antibody for 2 hours at 37°C. Nuclei were stained with DAPI for 15 minutes at 37°C. The sections were photographed using Olympus cellSens Entry.

### 2.8 Carotid artery wire ligation injury model

All experimental protocols were approved by the Ethics Committee of Tongji Medical College, Huazhong University of Science and Technology, and were performed in accordance with relevant institutional and national guidelines and regulations. In brief, the left carotid artery of C57BL/6 mice was ligated and the right carotid artery was performed sham‐surgery injury. Apatinib (10 mg/kg/day) was intraperitoneally injected to C57BL/6 mice for 14 days after carotid artery wire ligation. Six vehicle‐treated mice (150 μL of 5% DMSO i.p. injection) served as controls. After 14 days, mice were euthanized and injured vessels and sham‐surgery vessels were removed. After being fixed with 4% formaldehyde and embedded in paraffin, vessels were cut into sections. The sections were stained with haematoxylin‐eosin (H&E) and elastic Masson trichrome solutions. The intimal and medial thickness of each arterial section was assessed with program ImageJ. The mean intimal thickness, medial thickness and intima/media ratio were calculated.

### 2.9 Statistical analysis

All experiments were performed at least three times, and data are expressed as means ± SEM. Statistical significance was estimated by Student′s *t* test for the comparison of two groups and one‐way ANOVA for the comparison of several groups, and differences were considered significant at *P* < .05.

## 3 RESULTS

### 3.1 Apatinib inhibits intimal hyperplasia and vascular remodelling after vascular injury in vivo

**Figure 1:**
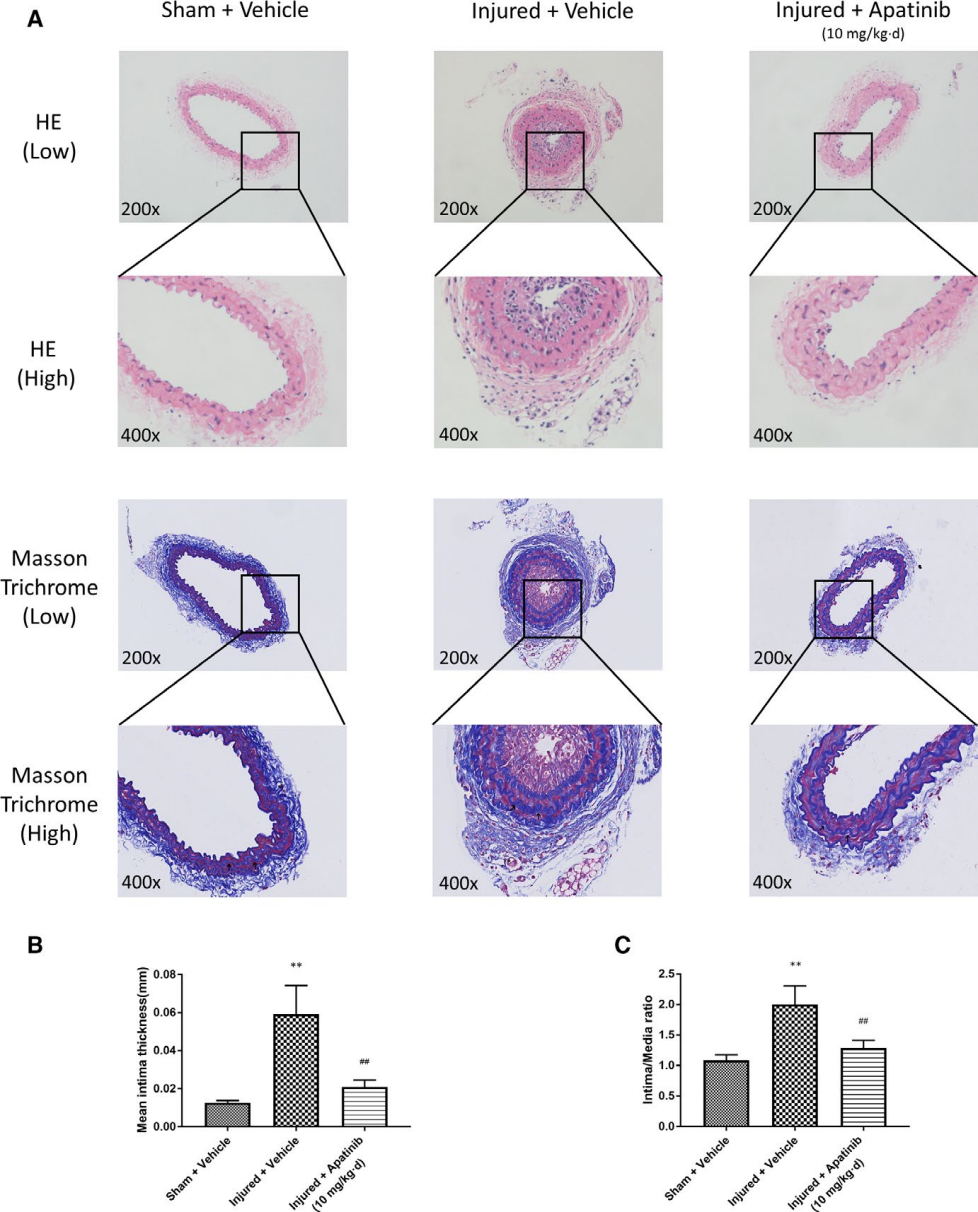
Effect of apatinib on intimal thickness induced by wire ligation injury for 14 days in vivo. A, C57BL/6 mice were treated with vehicle or apatinib for 14 days since the first day of being performed sham‐surgery injury or wire ligation injury. After 14 days of vehicle or apatinib administration, carotid arteries were harvested, fixed and embedded in paraffin. H&E‐stained and elastic Masson trichrome‐stained sections of all groups were shown. The images shown are representative of those obtained from 6 independent experiments. Arrowheads indicate elastic lamina. B, Measurement of mean intimal thickness. Data are represented as means ± SEM (n = 6 for each experimental group). **P < .01 versus the sham + vehicle group. ##*P* < .01 versus the injured + vehicle group. C, Quantification of intima/media ratio. Data are represented as means ± SEM (n = 6 for each experimental group). **P < .01 versus the sham + vehicle group. ##*P* < .01 versus the injured + vehicle group

Firstly, we used carotid artery wire ligation model to assess the effect of apatinib on intimal hyperplasia and vascular remodelling after vascular injury. We injected with apatinib intraperitoneally to C57BL/6 mice that had been performed wire ligation injury. Sham surgery and injection of vehicle served as controls. The sections stained with elastic Masson trichrome solutions were used to highlight the media. It is found that mice injected with vehicle developed a neointima which was notably alleviated in apatinib‐treated mice for 14 days after injury (Figure 1A). In two vascular injury groups, the treatment of apatinib exhibited a significant reduction in intimal thickness and intima to media ratio compared to the treatment of vehicle (Figure 1B,C). These results indicate that apatinib administration significantly diminish the arterial proliferative and migratory response to vascular injury.

### 3.2 Apatinib inhibits VSMC proliferation induced by PDGF‐BB

**Figure 2:**
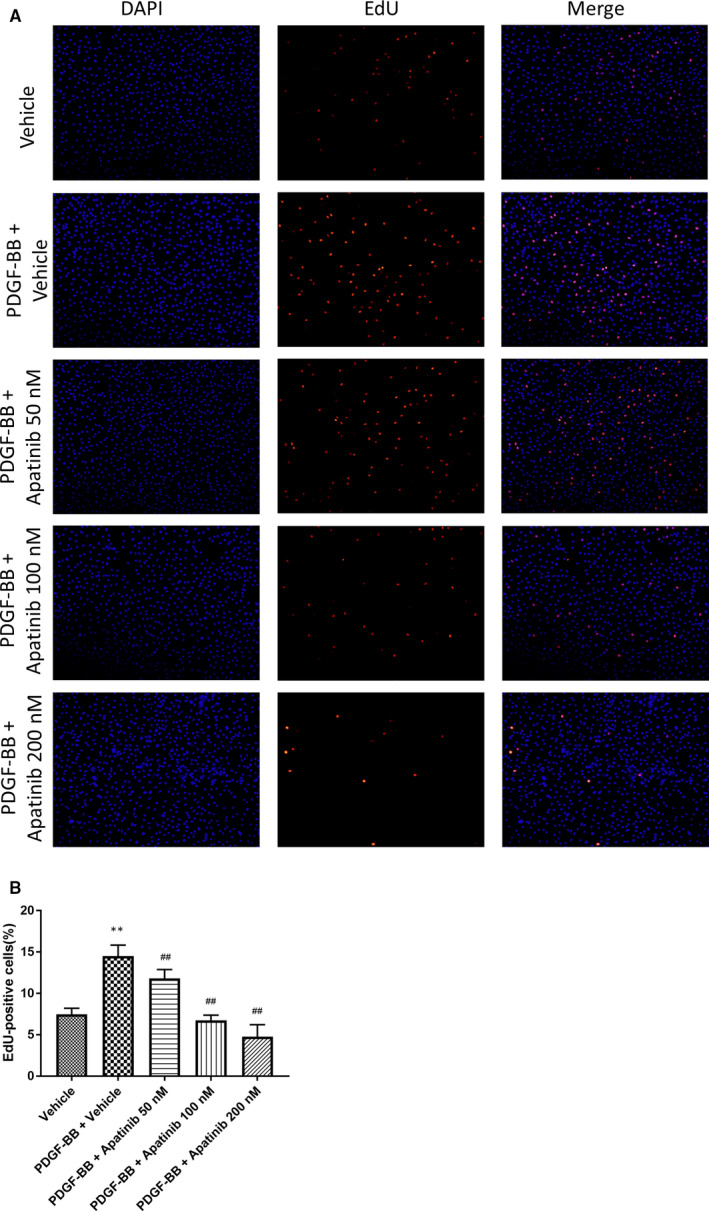
Effect of apatinib on VSMC proliferation induced by PDGF‐BB. A, VSMCs were stained with EdU (red) and DAPI (blue). B, Quantification of the proliferative cells. Data are represented as mean ± SEM (n = 5). **P < .01 versus the vehicle group. ##*P* < .01 versus the PDGF‐BB + vehicle group

**Figure 3:**
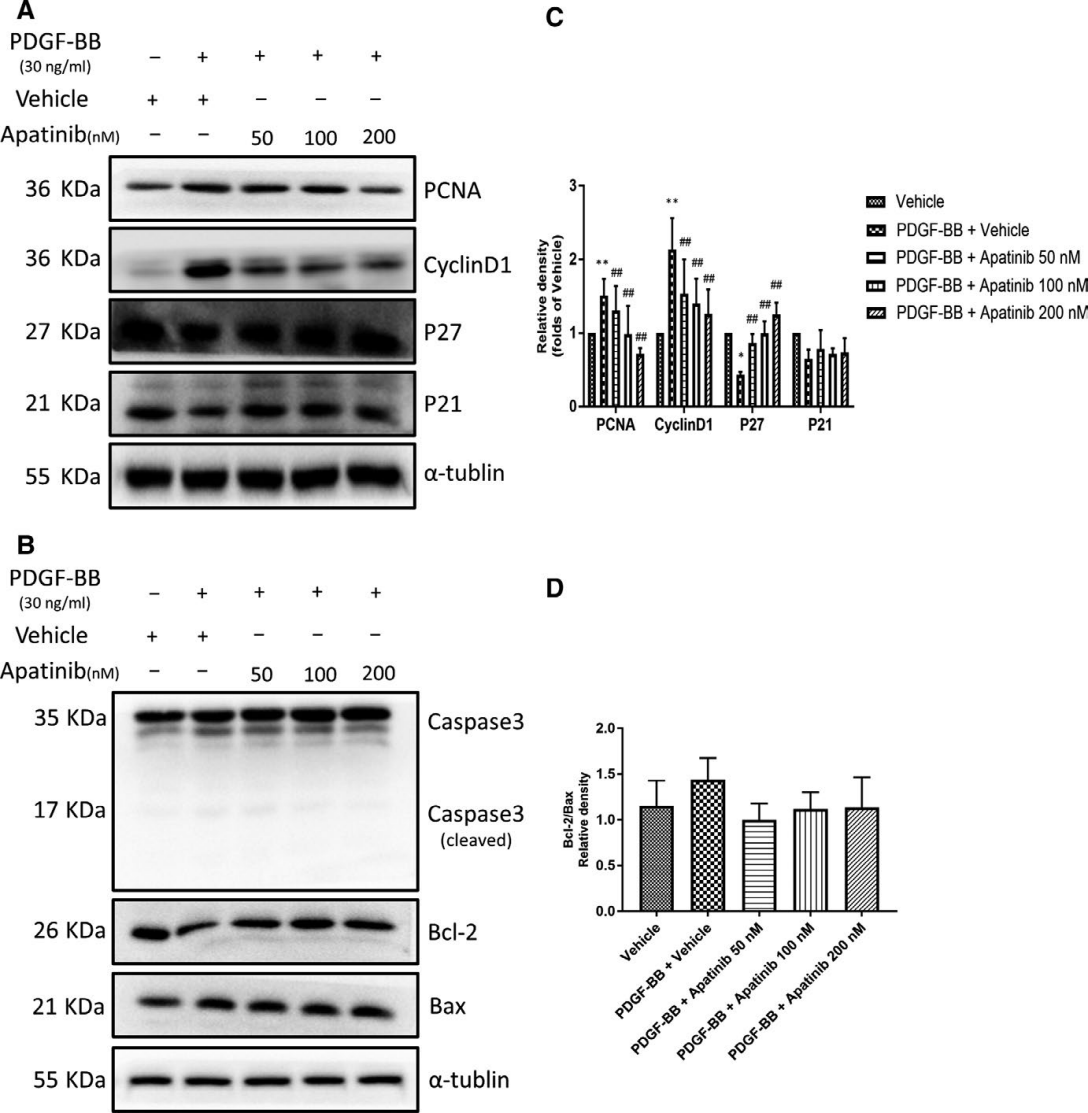
Effect of apatinib on the expression of cell proliferation and apoptosis marker genes. A, The protein level of PCNA, Cyclin D1, P27 and P21 were determined by Western blots. B, Quantification of the Western blots. Data are represented as mean ± SEM (n = 3). **P < .01 versus the vehicle group. ##*P* < .01 versus the PDGF‐BB + vehicle group. C, The protein level of caspase3 (cleaved), Bcl‐2 and Bax was determined by Western blots. D, Quantification of Bcl‐2/ Bax relative density using the Western blots. Data are represented as mean ± SEM (n = 3)

The proliferative and migratory abilities of VSMCs are regulated by lots of factors, including PDGF‐BB.^11^ Firstly, We examined the effect of apatinib on VSMC proliferation using EdU Incorporation Assay. The stimulation of PDGF‐BB (30 ng/mL) increased VSMC proliferation at 48 hours compared to the vehicle group. Apatinib significantly reduced VSMC proliferation induced by PDGF‐BB in a concentration‐dependent manner (Figure 2A,B). Meanwhile, the expression of cell proliferation marker genes including PCNA, Cyclin D1, P21, P27 was detected using Western blotting. Consistent with Figure 2, the expression of PCNA and Cyclin D1 increased, whereas P27 decreased with the stimulation of PDGF‐BB, apatinib blocked these effects. However, the protein level of P21 showed no significant change (Figure 3A,B). We also assessed caspase3 cleavage (from 35 to 17‐19 kDa), Bcl‐2 and Bax as apoptosis markers. Apatinib treatment did not change the expression of these genes (Figure 3C,D). These results suggest that apatinib inhibit VSMC proliferation induced by PDGF‐BB, and this inhibitive effect was not due to apoptosis.

### 3.3 Apatinib inhibits VSMC migration induced by PDGF‐BB

**Figure 4:**
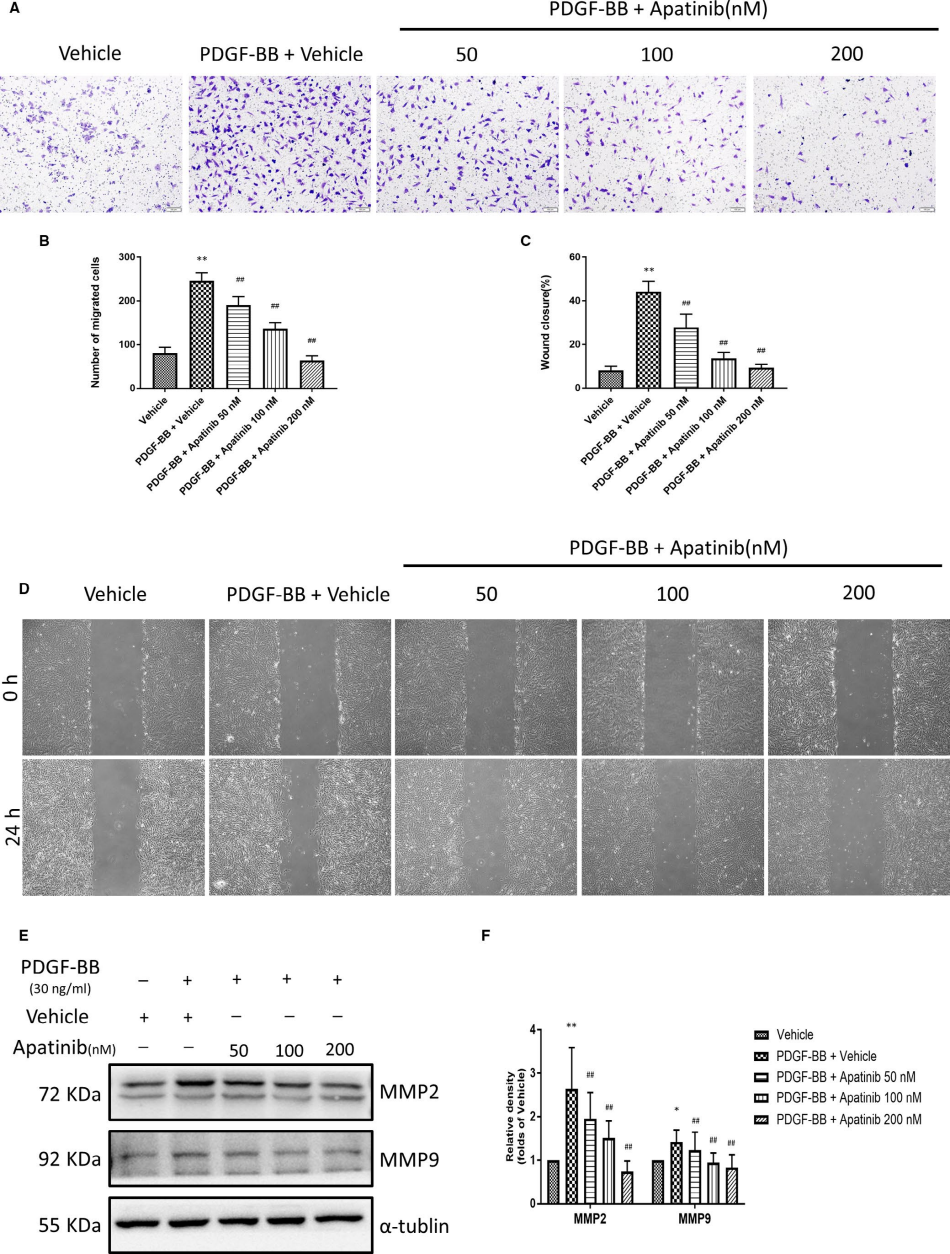
Effect of apatinib on VSMC migration induced by PDGF‐BB. A, Migrated VSMCs were photographed after being stimulated with PDGF‐BB (30 ng/mL) for 12 hours in the presence or absence of apatinib treatment. B, Quantification of migrated cells. Data are represented as mean ± SEM (n = 5). **P < .01 versus the vehicle group. ##*P* < .01 versus the PDGF‐BB + vehicle group. C, Cell monolayers were scratched and treated with vehicle or indicated concentrations of apatinib in the presence of PDGF‐BB (30 ng/mL) for 24 hours. D, Quantification of the area of wound closure (%). Data are represented as mean ± SEM (n = 4). **P < .01 versus the vehicle group. ##*P* < .01 versus the PDGF‐BB + vehicle group. E, The protein levels of MMP2 and MMP9 were detected by Western blots. F, Quantification of the Western blots. Data are represented as mean ± SEM (n = 3). *P < .05, **P < .01 versus the vehicle group. #*P* < .05, ##*P* < .01 versus the PDGF‐BB + vehicle group

Previous studies have shown that apatinib can significantly inhibit the migration of cholangiocarcinoma cell.^12^ PDFG‐BB is an effective growth factor which could increase VSMC migration as well.^11^ Therefore, we would like to know whether it could suppress the migratory ability of VSMC induced by PDGF‐BB. The migratory capacity was evaluated by wound healing assays and transwell cell migration assays. Before the stimulation of PDGF‐BB (30 ng/mL), VSMCs were preincubated with apatinib at indicated concentrations for 4 hours. We found that the stimulation of PDGF‐BB (30 ng/mL) increased VSMC penetration through the membrane significantly. However, apatinib reduced the number of migrated cells (Figure 4A,B). Meanwhile, wound healing test showed that apatinib inhibited cell migration induced by PDGF‐BB in a dose‐dependent manner (Figure 4C,D). In addition, the expression of MMP‐2 and MMP‐9, which are implicated to cell migration,^13^, ^14^ was detected using Western blotting, and we found that apatinib inhibited the expression of MMP2 and MMP9 effectively (Figure 4E,F). These results indicate that apatinib is an effective inhibitor of VSMC migration.

### 3.4 Apatinib inhibits VSMC phenotype dedifferentiation in vitro and in vivo

**Figure 5:**
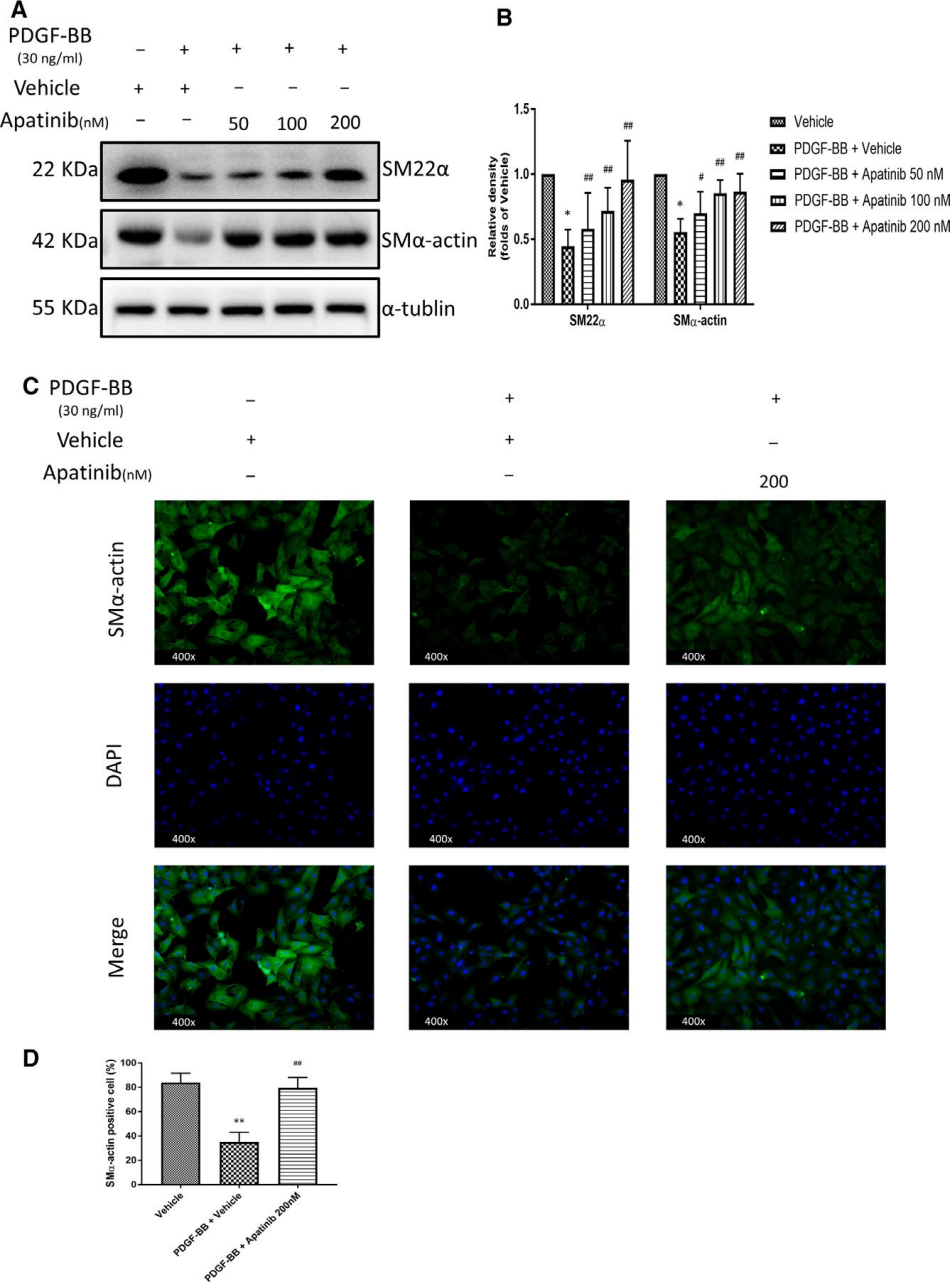
Effect of apatinib on VSMC dedifferentiation induced by PDGF‐BB in vitro. A, The protein levels of SMα‐actin, SM22α were determined by Western blots. B, Quantification of the Western blots. Data are represented as mean ± SEM (n = 3). *P < .05 versus the vehicle group. #*P* < .05, ##*P* < .01 versus the PDGF‐BB + vehicle group. C, Cells were stained with antibodies SMα‐actin (green); nuclei were stained with DAPI (blue). Each group was photographed within the same exposure time. D, Quantification of the SMα‐actin positive cells. Data are represented as mean ± SEM (n = 4). **P < .01 versus the vehicle group. ##*P* < .01 versus the PDGF‐BB + vehicle group

**Figure 6:**
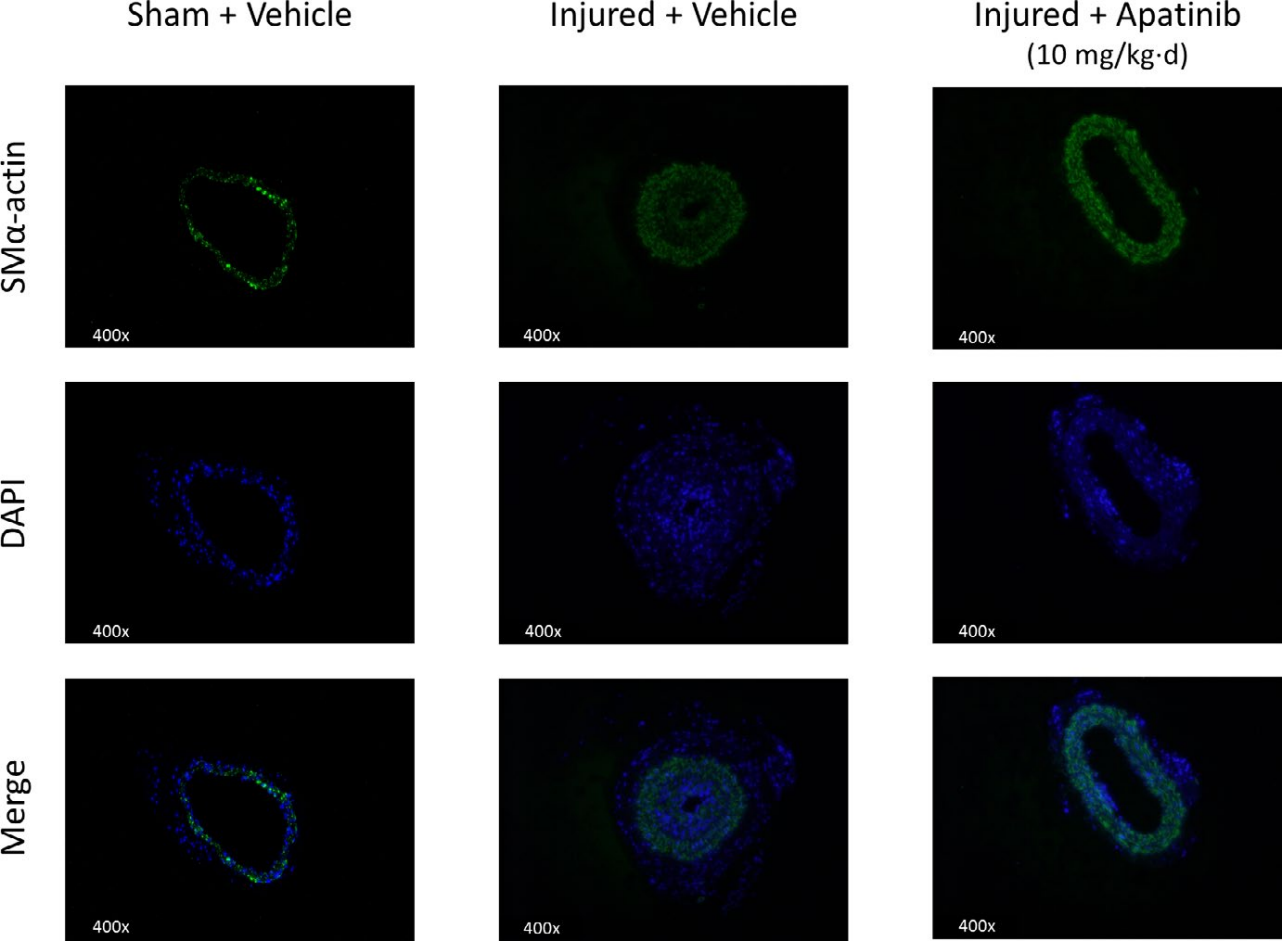
Effect of apatinib on VSMC dedifferentiation induced by PDGF‐BB in vivo. A, SMα‐actin staining in the injured or sham‐surgery carotid vessel walls. Cells were stained with antibodies SMα‐actin (green); nuclei were stained with DAPI (blue). Each group was photographed within the same exposure time

With the stimulation of PDGF‐BB, VSMC not only exhibited an increasing proliferation and migration, but also dedifferentiation.^15^ In order to investigate the effect of apatinib on phenotypic regulation of VSMC, we examined the effect of apatinib on VSMC dedifferentiation. VSMC dedifferentiation is characterized by a decreased expression of some contraction genes, including SMα‐actin and SM22α. VSMCs were pretreated with apatinib at different concentrations for 4 hours and stimulated with PDGF‐BB (30 ng/mL) for 48 hours. The expression of SMα‐actin and SM22α was detected by Western blotting. As is shown, PDGF‐BB reduced the protein levels of SMα‐actin and SM22α. However, apatinib pretreatment reversed the VSMC dedifferentiation (Figure 5A,B). Meanwhile, the cellular immunofluorescence analysis also confirmed that with the stimulation of PDGF‐BB, VSMCs exhibited elevated proliferation and the relative fluorescence intensity of SMα‐actin was decreased compared to the vehicle group; then, the maximum concentration of apatinib (200 nmol/L) was given could restore the fluorescence intensity of SMα‐actin(Figure 5C,D). The presence of SMα‐actin in the carotid artery wire ligation injury model was also visualized by immunofluorescence (Figure 6A). The expression of SMα‐actin was higher in apatinib‐treated mice compared to vehicle mice. These data indicate that apatinib inhibits dedifferentiation of VSMC in vitro and in vivo.

### 3.5 Apatinib inhibits PDGFR‐β signalling induced by PDGF‐BB in VSMCs and cell line A7r5

**Figure 7:**
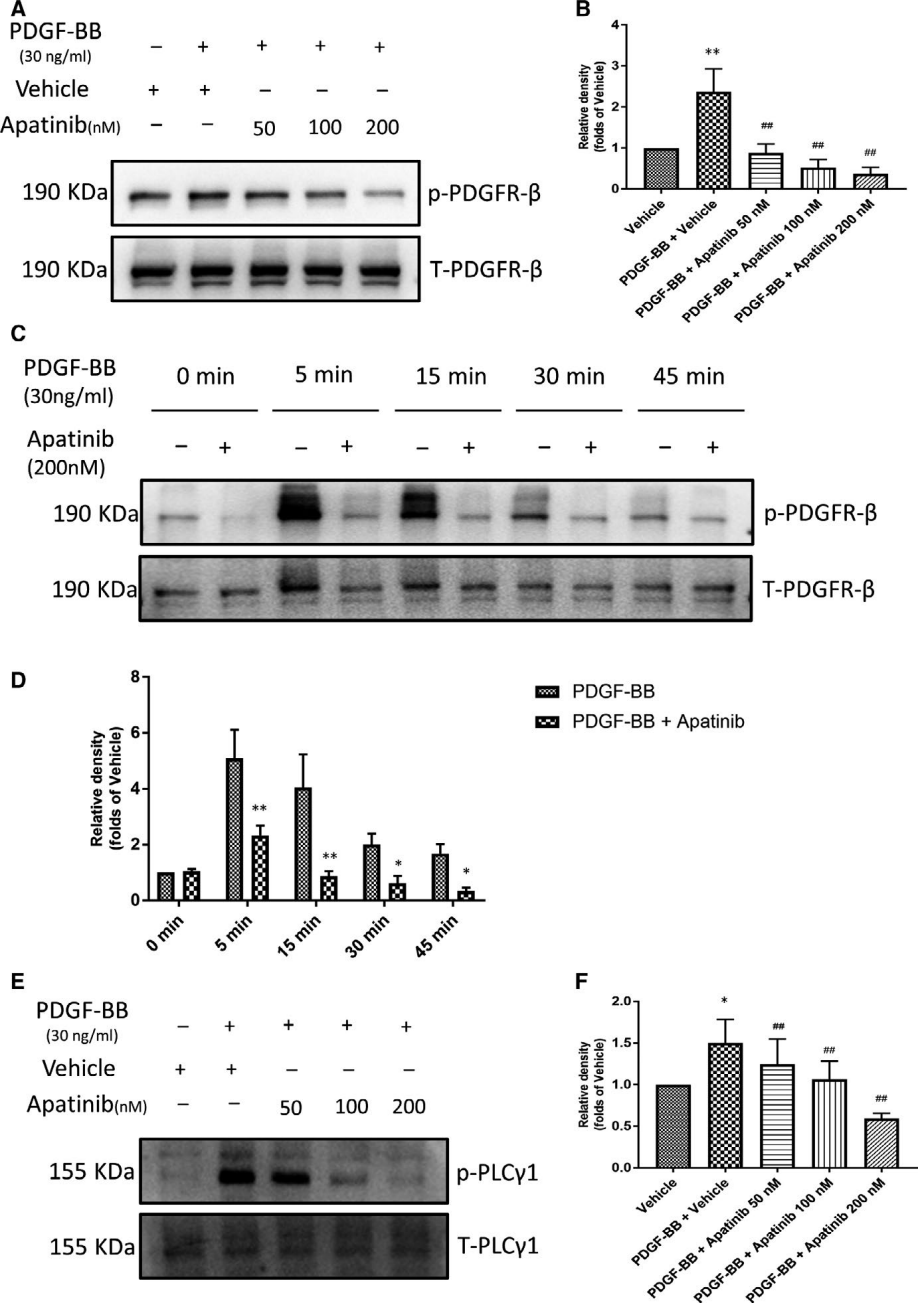
Effect of apatinib on the phosphorylation of PDGFR‐β in VSMCs and cell line A7r5. A, The protein levels of phospho‐PDGFR‐β, PDGFR‐β in VSMCs were determined using Western blot. B, Quantification of the Western blots. Data are represented as mean ± SEM (n = 3). *P < .05 versus the vehicle group. #*P* < .05, ##*P* < .01 versus the PDGF‐BB + vehicle group. C, The protein levels of phospho‐PDGFR‐β, PDGFR‐β in cell line A7r5 were determined using Western blots. D, Quantification of the Western blots. Data are represented as mean ± SEM (n = 3). *P < .05, **P < .01 versus the vehicle control. E, The protein levels of phospho‐PLC‐γ1, PLC‐γ1 in VSMCs were determined using Western blots. F, Quantification of the Western blots. Data are represented as mean ± SEM (n = 3). **P < .01 versus the vehicle group. ##*P* < .01 versus the PDGF‐BB + vehicle group

PDGFR‐β activation by PDGF‐BB plays a key role in dysregulation of VSMC proliferation and migration.^16^ The previous study confirmed that apatinib could suppress the phosphorylation of PDGFR‐β in NIH‐3T3 cells with the stimulation of PDGF‐BB.^9^ It is reasonable to presume apatinib inhibits vascular remodelling after vascular injury by targeting PDGFR‐β. Therefore, we tested if apatinib could suppress the phosphorylation of PDGFR‐β in VSMCs. After pretreatment with apatinib at different concentrations for 4 hours, it is found that apatinib could inhibit the phosphorylation (Tyr751) of PDGFR‐β on the surface of VSMC membrane in a dose‐dependent manner (Figure 7A,B). To further confirm the inhibitory effect of apatinib on PDGFR‐β phosphorylation, we use rat vascular smooth muscle cell line A7r5 to verify this phenomenon. A7r5 were pretreated with apatinib at maximum concentration (200 nmol/L) for 1 hours and then stimulated with PDGF‐BB for a specified duration. As observed in A7r5, PDGFR‐β was well activated at 5 minutes, whereas preincubation of apatinib significantly inhibited PDGFR‐β at all observed time points (5, 15, 30, 45 minutes) and this inactivation was not due to a decrease in total protein expression levels (Figure 7C,D). Meanwhile, we detected the expression of tyrosine kinase PLC‐γ1 which is directly conjugated to PDGFR^17^; it is found that with the stimulation of PDGF‐BB, the expression of PLC‐γ1 remains stable, whereas the phosphorylation of PLC‐γ1 was suppressed by apatinib, showing the same trends with p‐PDGFR‐β (Figure 7E,F), which indicates that apatinib attenuates the pathological activation of VSMCs through the suppression of PDGFR‐β.

### 3.6 Apatinib inhibits MAPK signalling pathway

**Figure 8:**
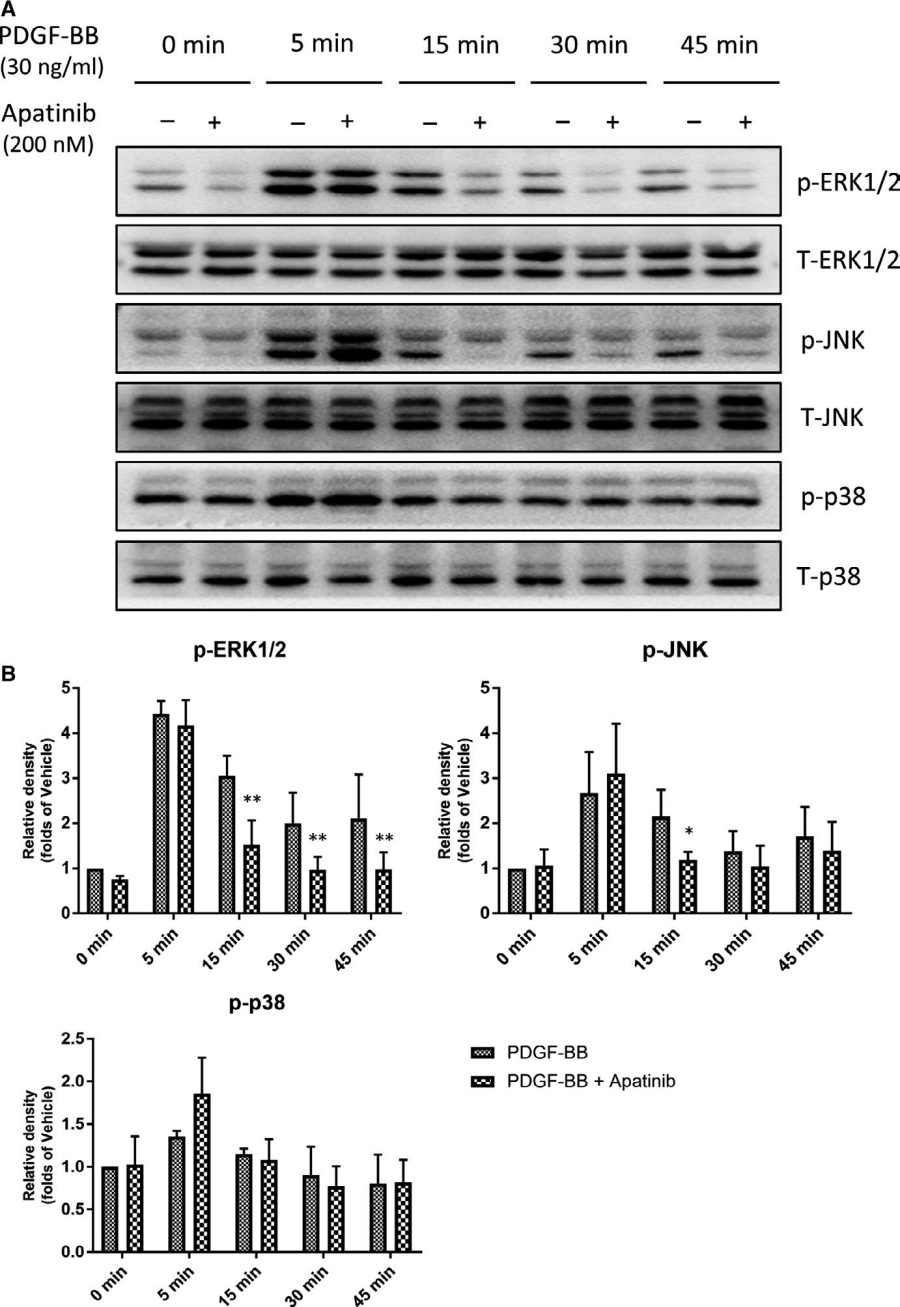
Effect of apatinib on the MAPK signalling pathway in cell line A7r5. A, The protein levels of phospho‐ERK1/2, ERK1/2, phospho‐p38, p38, phospho‐JNK and JNK in cell line A7r5 were determined using Western blots. B, Quantification of the Western blots. Data are represented as mean ± SEM (n = 3). *P < .05 versus the vehicle control

MAPK signalling pathway, as one of the most significant downstream pathways of PDGFR‐β‐mediated signalling, plays significant role in some aspects, including cell proliferation and migration.^5^ VSMC dedifferentiation induced by PDGF‐BB involves the activation of MAPK signalling and the inhibition of ERK1/2, JNK and p38 abolishes PDGF‐BB‐induced dedifferentiation.^18^ Therefore, we investigated the role of MAPK signalling pathway in the ability of apatinib to modulate the VSMC phenotype in response to PDGF‐BB. Phosphorylation of ERK1/2, JNK and p38 increased at 5 minutes after PDGF‐BB stimulation, and the phosphorylation of ERK1/2 was eliminated by apatinib (200 nmol/L) at 15, 30, 45 minutes. Although the inhibitory effect of apatinib on phosphorylated JNK was statistically significant only at 15 minutes, there was still a trend of inhibition at 30 and 45 minutes during the observation. However, the phosphorylated p38 was not inhibited by apatinib (Figure 8A,B). No significant change was observed in the total protein expression of these signalling molecules in the presence or absence of apatinib during the stimulation of PDGF‐BB. These data suggest that ERK1/2 and JNK signalling pathways are involved in the regulation of apatinib on VSMC phenotype in response to PDGF‐BB.

## 4 DISCUSSIONS

The characteristic of our study is that a small‐molecule tyrosine kinase inhibitor was newly applied to a potential therapeutic field. We firstly demonstrated that the treatment of apatinib significantly inhibited intimal hyperplasia and vascular remodelling in vivo and PDGF‐BB‐induced VSMC phenotypic transformation in vitro. Our data confirmed the suppressive effects of apatinib on VSMC proliferation, migration and dedifferentiation induced by PDGF‐BB. Besides, neointimal hyperplasia caused by arterial wire ligation injury in vivo was also suppressed by apatinib administration.

VSMC phenotype switches from contractile phenotype to proliferative and migratory synthetic phenotype after being stimulated by injury, inflammation or high level of glucose.^19^ VSMC phenotypic transformation is a basic step in VSMC proliferation and migration, which are indispensable in the development of atherosclerosis or restenosis after angioplasty. Drugs that ameliorate vascular remodelling can inhibit the proliferation and migration of VSMC. A previous study has shown that apatinib treatment can reduce the expression of Cyclin D1 in osteosarcoma cell line KHOS cells and MG63 cells in inhibiting cell cycle.^20^ Our study suggested that apatinib inhibits VSMC proliferation with a decreased protein level of Cyclin D1. Apatinib promotes apoptosis in osteosarcoma and anaplastic thyroid cancer, respectively, in previous studies.^20^, ^21^ However, we found apatinib inhibits VSMC proliferation at maximum concentration (200 nmol/L) in our study but it did not induce apoptosis.

Apatinib was firstly demonstrated to inhibit the phosphorylation of PDGFR‐β in NIH‐3T3 cells induced by PDGF‐BB. Our data confirmed this inhibitive effect in VSMCs and cell line A7r5, respectively. PDGF‐BB, as one of the most potent isoforms in the PDGF family, has been well demonstrated in PDGF‐BB‐treated dedifferentiation of VSMCs.^22^ It exists in platelets and could be formed in endotheliocyte, smooth muscle cell as well as monocyte‐derived macrophages. Previous studies suggest endogenous PDGF plays an important role in the accumulation of neointimal smooth muscle cells caused by balloon injury and restenosis after angioplasty.^23^ Sunitinib is another small‐molecule TKI with antiangiogenic and antitumour effects. Oral administration of sunitinib (10 mg/kg/day) attenuates the pathological activation of VSMC through the inhibition of PDGF signalling which activates VSMC in arterial remodelling. ^24^ In D. Loau's study,^25^ it is found that the mean concentration of apatinib in S‐D rat plasma samples reached its highest (382.3 ± 46.7 ng/mL) at 2 hours and decreased to its lowest (5 ng/mL) at 12 hours after oral administration at the dose of 40 mg/kg. This is the reference for our study to choose the dosage in vivo.

The binding of PDGF‐BB to PDGFR results in the phosphorylation of PDGFR‐β tyrosine residues and the activation of PLC‐γ1 tyrosine residues, which activates many downstream signalling molecules, such as MAPK pathway including ERK1/2, p38 and JNK pathways.^26^ PLC‐γ forms a complex with PDGF receptors, which leads to the phosphorylation of PLC‐γ.^17^ Inhibition of these signalling pathways can postpone the progression and development of proliferative vascular diseases. Consistent with previous studies, it was found that PDGF‐BB triggered VSMCs dedifferentiation and simultaneously induced the phosphorylation of ERK1/2, p38 and JNK.^27^, ^28^ These results indicated that the inhibition of PDGFR‐β, ERK1/2, JNK signalling may be involved in the effects of apatinib on VSMC phenotype modulation. Therefore, modulation of the related signalling pathways may be an essential pharmacological point for the prevention of vascular proliferative diseases. There are other essential pathways^27^ involved in regulating phenotypic transformation of VSMCs, and we will examine the effect of apatinib on them in subsequent experiments.

In conclusion, our study suggests that apatinib modulates VSMC phenotypic transformation by inhibiting the pathological activation of VSMCs through the suppression of multiple pathways including PDGFR‐β and its downstream MAPK signalling pathway. Apatinib may have a beneficial effect on cardiovascular diseases.

## CONFLICT OF INTEREST

The authors confirm that there are no conflicts of interest.

## AUTHOR CONTRIBUTIONS

Kai Huang conceived and designed the experiments. Wenchao Shao performed the experiments and Xiaoguang Li drafted the manuscript. Jiangtong Peng and Siyuan Fan participated in discussions of data analysis. Minglu Liang provided critical suggestions and revised the manuscript. All authors gave final approval.

## Supporting information

Figure S1

## Data Availability

All data generated or analysed during this study are included in this article.
