# Supplementary figures and images for: Apatinib attenuates phenotypic switching of arterial smooth muscle cells in vascular remodelling by targeting the PDGF Receptor‐β
Source: J Cell Mol Med. 2020 Jul 22;24(17):10128–39. doi: 10.1111/jcmm.15623 (PMC7520274; doi:10.1111/jcmm.15623)

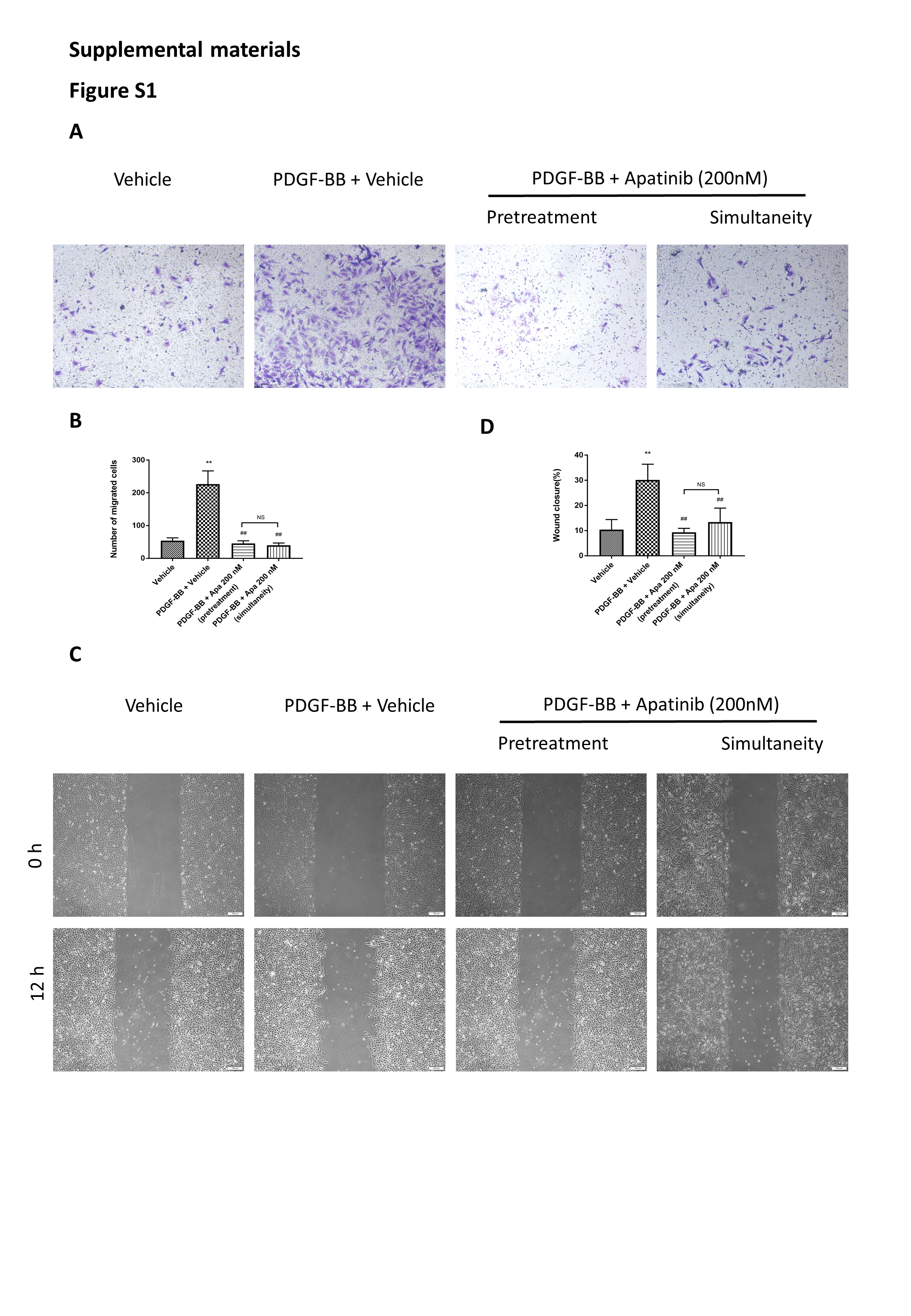

Supplement: Supplementary file 1 — Figure S1 [file JCMM-24-10128-s001.tif]
